# The overlooked complexity of avian brood parasite–host relationships

**DOI:** 10.1111/ele.14062

**Published:** 2022-06-28

**Authors:** James A. Kennerley, Marius Somveille, Mark E. Hauber, Nicole M. Richardson, Andrea Manica, William E. Feeney

**Affiliations:** ^1^ Department of Zoology University of Cambridge Cambridge UK; ^2^ Centre for Biodiversity and Environment Research University College London London UK; ^3^ Department of Evolution, Ecology, and Behavior School of Integrative Biology University of Illinois at Urbana‐Champaign Urbana Illinois USA; ^4^ Department of Biosciences Durham University Durham UK; ^5^ Department of Behavioural Ecology and Evolutionary Genetics Max Planck Institute for Ornithology Starnberg Germany

**Keywords:** bird, brood parasitism, coevolution, cowbird, cuckoo, ecology, evolution, multispecies interactions

## Abstract

The relationships between avian brood parasites and their hosts are widely recognised as model systems for studying coevolution. However, while most brood parasites are known to parasitise multiple species of host and hosts are often subject to parasitism by multiple brood parasite species, the examination of multispecies interactions remains rare. Here, we compile data on all known brood parasite–host relationships and find that complex brood parasite–host systems, where multiple species of brood parasites and hosts coexist and interact, are globally commonplace. By examining patterns of past research, we outline the disparity between patterns of network complexity and past research emphases and discuss factors that may be associated with these patterns. Drawing on insights gained from other systems that have embraced a multispecies framework, we highlight the potential benefits of considering brood parasite–host interactions as ecological networks and brood parasitism as a model system for studying multispecies interactions. Overall, our results provide new insights into the diversity of these relationships, highlight the stark mismatch between past research efforts and global patterns of network complexity, and draw attention to the opportunities that more complex arrangements offer for examining how species interactions shape global patterns of biodiversity.

## Graphical abstract

The relationships between avian brood parasites and their hosts are widely recognised as model systems for studying coevolution. However, while most brood parasites are known to parasitise multiple species of host and hosts are often subject to parasitism by multiple brood parasite species, the examination of multispecies interactions remains rare. By considering brood parasite–host interactions as ecological networks, we outline the disparity between patterns of network complexity and past research emphases, and highlight the potential benefits of considering brood parasitism as a model system for studying multispecies interactions.

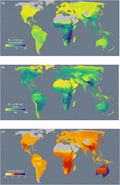

## INTRODUCTION

Coevolution—the process of reciprocal evolutionary change driven by cycles of adaptation and counter‐adaptation in pairs of interacting species—often produces stunning yet tractable phenotypes in the involved species (Dawkins & Krebs, 1979). As such, these exchanges have become a dominant feature of research aiming to explain the world's biodiversity (Betts et al., 2016; Feeney et al., 2014; Strauss & Irwin, 2004). Examples of coevolution are found throughout the natural world and include interactions between iconic duos, such as predatory bats which have evolved finely tuned sensory systems to hunt their moth prey and the respective moths which have, in turn, evolved to jam their predators' echolocation (Corcoran et al., 2009); and figs (*Ficus* sp.) that can only be pollinated by certain species of fig wasps (Agaonidae), which in turn rely on the figs to harbour their eggs and larvae as they mature (Machado et al., 2005). The examination of evolutionary exchanges, such as these, has underpinned insights into the evolution of adaptations within species (Paterson et al., 2010; Schulte et al., 2010), patterns of phenotype evolution across species (Lewis et al., 2019), and the evolution of species outright (Smith & Benkman, 2007; Sorenson et al., 2003). However, species interactions rarely occur in isolation, and indirect effects that arise from secondary relationships can play an important role in determining the outcome of these exchanges (Castledine et al., 2020; Guimarães et al., 2017; Toju et al., 2017). For instance, the removal of herbivores can lead to the breakdown of mutualisms between ants and plants (Palmer et al., 2008), high rates of nest predation can lead to the relationships between obligate brood parasitic (*herein* ‘brood parasitic’) cuckoos and some of their hosts shifting from parasitic to mutualistic (Canestrari et al., 2014), and the pressure imposed on fishes by their predators can explain the recurrent convergent evolution of protection mutualisms with toxic anemones (Feeney et al., 2019). Studies such as these highlight that the incorporation of additional layers of complexity remains a key challenge to understanding how species interactions operate and shape biodiversity (Thompson, 2005).

Early calls to study coevolution at a community level led Janzen (1980) to coin the term ‘diffuse coevolution’ which extended on the classic approach of studying a pair of species, to instead consider how guilds of species interact. ‘Diffuse coevolution’ has, however, been described as an inadequate term that often fails to fully capture the importance of ecological interactions (Iwao & Rausher, 1997; Lomáscolo et al., 2019) which, in part, is attributable to the traditional assumption that evolution and ecology act independently (Urban et al., 2020). In recent years, there has been recognition of the necessity to integrate ecology and evolutionary biology in order to understand the influence of interactions between two or more species across different spatial and temporal scales (Medeiros et al., 2018; Urban et al., 2020). Acknowledging the synergy between these two disciplines, this approach has led to widespread usage of the ‘multispecies’ descriptor (e.g. Bittleston et al., 2016; Roth‐Monzón et al., 2020) to refer to aspects of ecological networks which, in turn, has led to significant breakthroughs in our understanding of how biotic interactions influence evolutionary processes and affect biodiversity. For instance, experiments by Betts et al. (2018) found that bacterial hosts exhibited a higher rate of molecular evolution and diversification when exposed to a greater diversity of viral parasites; and Woodward et al. (2012) showed how climate change can alter the structure and composition of food webs, with larger and rarer species being disproportionately affected. With studies highlighting the benefits of a multispecies approach, some areas of research, such as the study of pollination and herbivory (Bascompte et al., 2006; Fox, 1988; Ramos & Schiestl, 2019) have embraced this line of thinking while other areas, such as the study of parasite–host interactions, have not (Betts et al., 2016).

**FIGURE 1:**
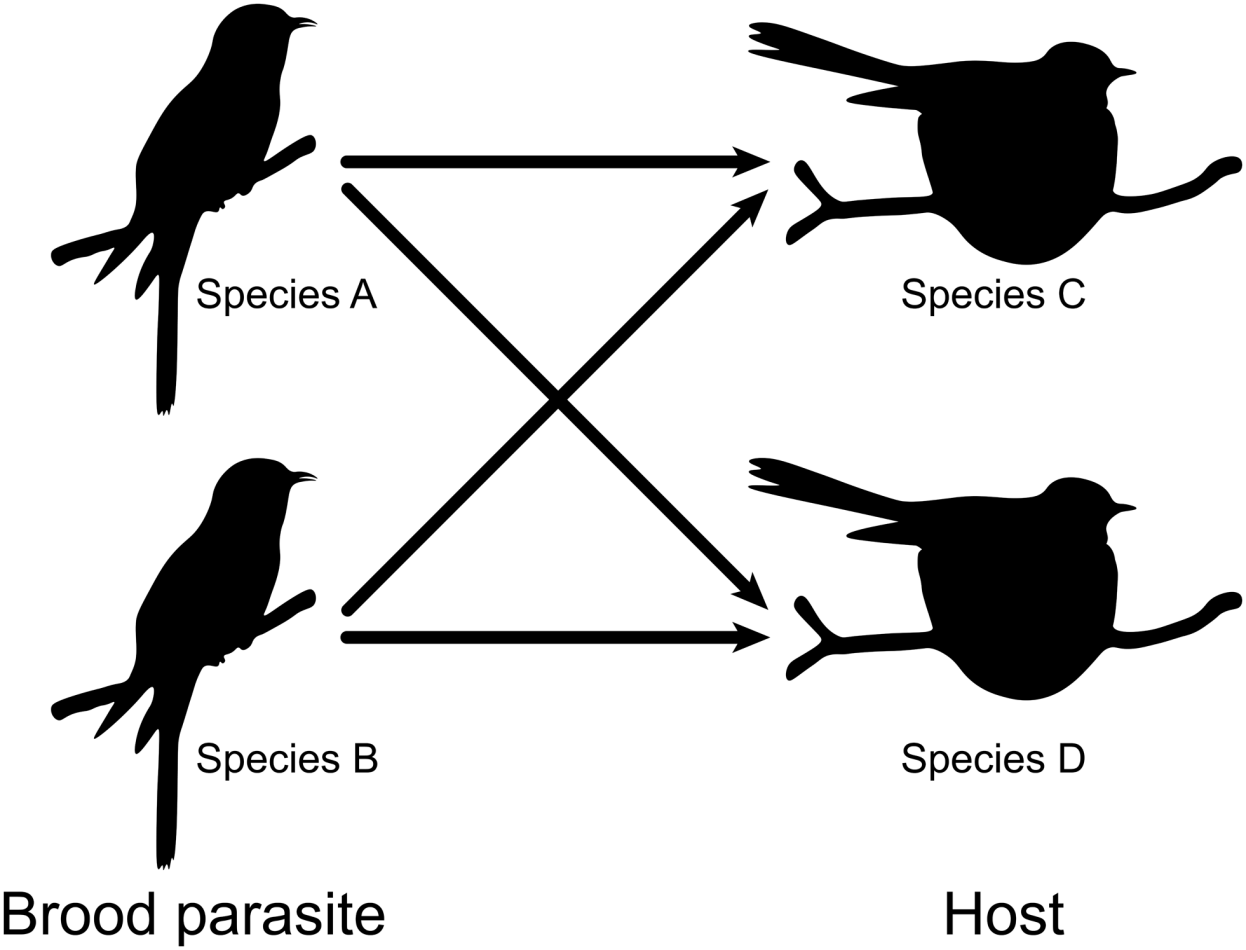
A diagram depicting the networks considered in this study: One‐to‐one (e.g. species A → C); one‐to‐many (e.g. species A → C and D); many‐to‐one (e.g. species A and B → C); and many‐to‐many (e.g. species A and B → C and D)

The relationships between obligate avian brood parasites and their hosts has been a source of fascination for millennia (Lai, 1998; Wentworth, 1910) and, over the past half century, they have become model systems for studying coevolutionary processes (Feeney et al., 2014; Payne, 1977; Rothstein, 1990). Brood parasites lay their eggs in the nests species of other birds and foist the cost of parental care onto their hosts. Hosting a parasite is costly, which selects for defensive adaptations in hosts and reciprocal offensive adaptations in brood parasites that can extend across all stages of the host's breeding cycle (Brooke & Davies, 1988; De Mársico et al., 2012; Langmore et al., 2003; Welbergen & Davies, 2009). Early studies on the Brown‐headed Cowbird (*Molothrus ater*) and its hosts in North America (Rothstein & Robinson, 1998), and the Common Cuckoo (*Cuculus canorus*) and its hosts in Europe (Stoddard & Kilner, 2013) typically focused on understanding pairwise interactions (i.e. one brood parasite and one host). This work led to major advances in our understanding of how coevolution and species interactions operate and shape morphological, sensory and behavioural aspects of host and brood parasite diversity (e.g. Čapek et al., 2010; Caves et al., 2015; Davies & Brooke, 1988; Feeney et al., 2012; Geltsch et al., 2016; Gloag et al., 2013; Grim, 2007; Kilner et al., 2004; Langmore et al., 2003; Moksnes & Røskaft, 1989; Soler et al., 2003; Spottiswoode & Stevens, 2010; Tanaka & Ueda, 2005; Thorogood & Davies, 2012; Yang et al., 2010). Yet, with many of the world's brood parasites and hosts overlapping in their distributions (Feeney et al., 2013; Liang et al., 2017), pairwise interactions often form part of a much more complex network involving brood parasites targeting more than one species of host, and hosts being parasitised by more than one species of brood parasite (Figure 1). Despite the continuing focus on brood parasite–host systems as models for studying coevolution in a pairwise framework, recent studies have demonstrated that viewing these interactions using a multispecies framework can provide novel insights into evolutionary processes (e.g. Antonson et al., 2020; Hanley et al., 2021; Medina & Langmore, 2016).

Here, we aim to extend beyond the traditional coevolutionary framework that has characterised past research and examine the extent to which brood parasitism may be used as a novel model system to study multispecies interactions. To do this, we first quantify past research on avian brood parasitism, including the species, the geographic localities and the complexity of the ecological relationships that have been studied, as well as potential changes in research foci over time. Next, using information on all known brood parasite–host relationships, we conduct a global analysis of brood parasite–host networks to examine the extent to which multispecies interactions exist and to identify global hotspots of network complexity. Finally, by drawing on insights gained from other fields that have advanced our understanding of multispecies interactions (e.g. Betts et al., 2018; Bramon Mora et al., 2020; Roth‐Monzón et al., 2020), we discuss potential opportunities to use brood parasitism as a model system for studying multispecies interactions.

## MATERIALS AND METHODS

### Patterns of past research

To explore patterns of past research we used the *Web of Science* to conduct a comprehensive search for peer‐reviewed papers focussing on obligate avian brood parasitism. We applied the following search terms across ‘All Databases’ covering the entire timespan of the database as of 26 September 2020:

$$\mathrm{TS}=\left(\text{brood}\;{\text{parasit}}^{*}\right)\;\text{AND}\;\mathrm{TS}=\left({\text{avian}}^{*}\;\text{OR}\;{\text{bird}}^{*}\right).$$

This search yielded 3782 returns. We filtered out papers that we could not access, either because the libraries at the University of Cambridge, University of Illinois at Urbana‐Champaign or the Max Planck Institute for Ornithology do not subscribe to the journal, or the citation information returned by *Web of Science* was incomplete or ambiguous (*n* = 221). We manually removed studies that were not relevant to obligate avian brood parasitism (*n* = 1684) or were supplementary materials to a paper already represented in the search (*n* = 67). For each of the remaining papers (*n* = 1810), we read through the article to extract information on the year of study, the species of brood parasite(s) and host(s) involved, and the latitude and longitude of the field site(s). For studies where field sites were listed but geographic coordinates were not provided, we either obtained coordinates from another study involving the same field site or located the field site using results from *Google* searches and the software, *Google Earth*. Studies were placed into one of six study categories: ‘field‐based’, ‘laboratory‐based’, ‘museum and/or collection‐based’, ‘molecular and/or genomic’, ‘review and/or meta‐analysis’ and ‘theoretical and/or computational’. The complexity of the system examined in each paper was also scored using four categories: one‐to‐one (one brood parasite and one host), one‐to‐many, many‐to‐one and many‐to‐many (Figure 1). Theoretical hosts (i.e. species predicted to be a host but for which there is no evidence) and species that were not native to the study area were excluded from the dataset along with their respective field sites. All percentages presented are rounded to the nearest whole number.

Using these data, we explored how the number of studies changed through time for each network type. We also investigated how field sites were distributed across the world and how this changed through time. To do this, we rounded geographic coordinates of field sites to the nearest 0.5 degrees latitude and longitude to eliminate duplicate entries of the same field site, which were assigned slightly different coordinates in their respective studies. We then plotted study sites on a world map to compare geographic patterns of research with the distributions of brood parasites and hosts, and global patterns of network complexity.

### Global patterns of brood parasite–host network complexity

Using Lowther (2012, 2017, 2019a, 2019b, 2019c, 2019d) and the studies that we reviewed for the analysis of past research, we compiled a dataset of all brood parasites and each of their known hosts, based on Handbook of the Birds of the World and BirdLife International (2019) taxonomy. There were no documented hosts for 21 species of brood parasites (see Table S1), so these species were excluded from further analyses.

To map global patterns of network complexity, we first overlaid a grid of equal‐area hexagons (ISEA3H resolution 7; hexagon area ~23,323 km^2^; Sahr et al., 2003) onto a global map of coastline boundaries to obtain 7483 land hexagons. We then intersected BirdLife's species native breeding range maps (BirdLife International and Handbook of the Birds of the World, 2017) for all brood parasite and host species with the global grid of land hexagons to obtain presence–absence data for each species in each hexagon. Next, we used the brood parasite–host interaction dataset to compile a list of pairwise interactions between the brood parasites and their respective hosts for each land hexagon. Using these data we constructed a brood parasite–host interaction network for each land hexagon. We term these networks ‘potential networks’ as they are based on relationships between the brood parasites and hosts that have been documented within the species' native range but not necessarily within the region bounded by the respective land hexagon. We then calculated the linkage density of each potential network (i.e. the number of interactions divided by the total number of species), a commonly used descriptor of network complexity (Bersier et al., 2002). For instance, the lowest possible linkage density (0.5) can refer to a simple network comprising one parasite, one host and one interaction (i.e. 1 interaction/2 species = 0.5; illustrated in Figure 1) whereas a linkage density of 1.0 can refer to a more complex network comprising two parasites, two hosts and four interactions (i.e. 4 interactions/4 species = 1.0; illustrated in Figure 1). With each hexagon assigned a value of linkage density based on its potential brood parasite–host interaction network, these data were used to construct a heatmap of brood parasite–host network complexity. All analyses were conducted using the statistical software R (R Core Team, 2020), and the package ‘bipartite’ (Dormann et al., 2008) was used to calculate linkage density, which is presented to one decimal place. To investigate whether network complexity could be predicted by brood parasite or host richness, or overall bird species richness, we fitted ordinary least squares models (OLSs) and simultaneous autoregressive models (SARs), a generalisation of linear models that allow the explicit inclusion of spatial autocorrelation (Kissling & Carl, 2008), with the autoregressive process introduced in the error term (error‐SAR). The spatial weight matrices of the SARs were calculated with a neighbourhood distance of 200 km and were row‐standardised (Kissling & Carl, 2008).

## RESULTS

### Patterns of past research

**FIGURE 2:**
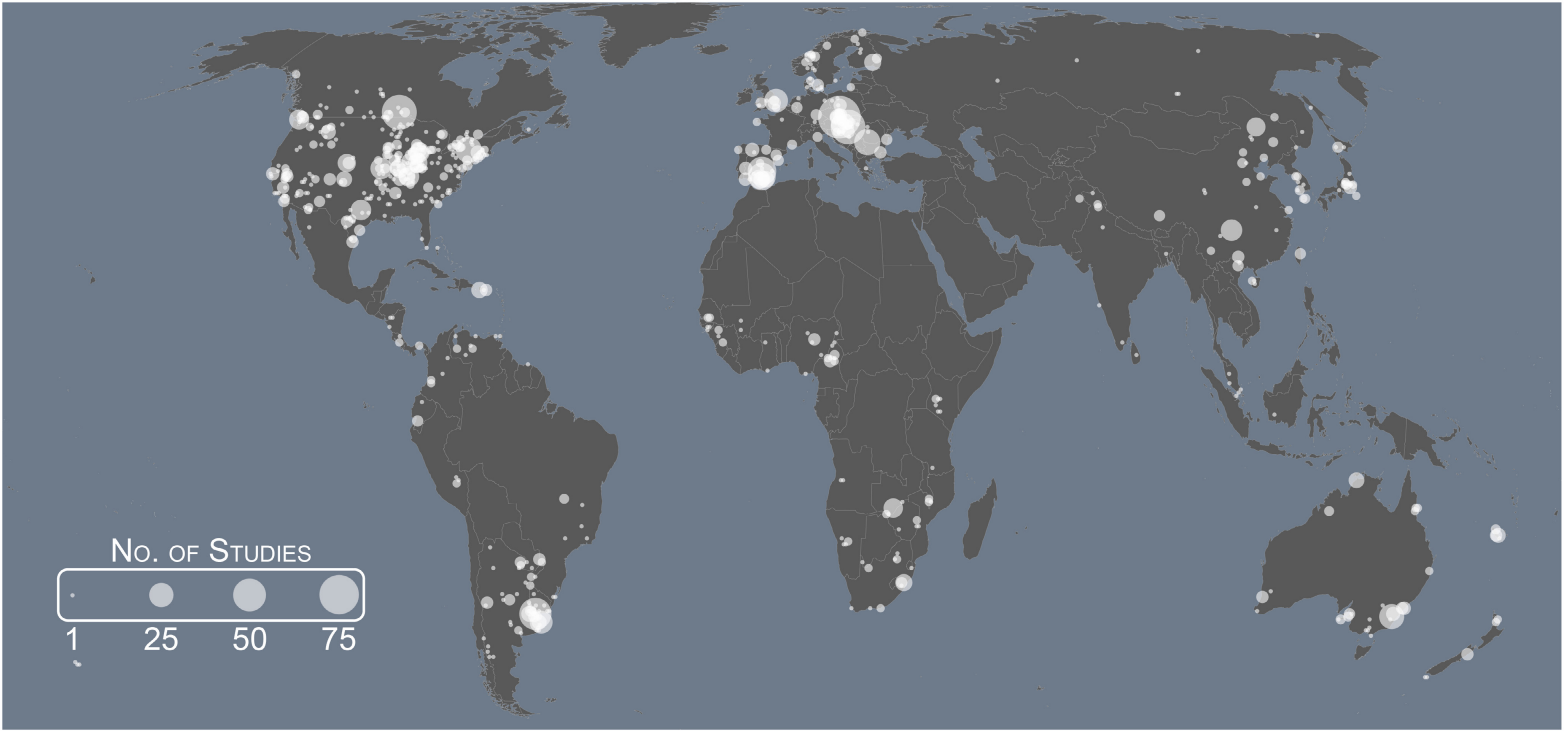
World map depicting field site locations utilised in brood parasitism studies. The size of each bubble correlates with the number of studies conducted at each field site, with larger bubbles indicating that more studies were conducted

**FIGURE 3:**
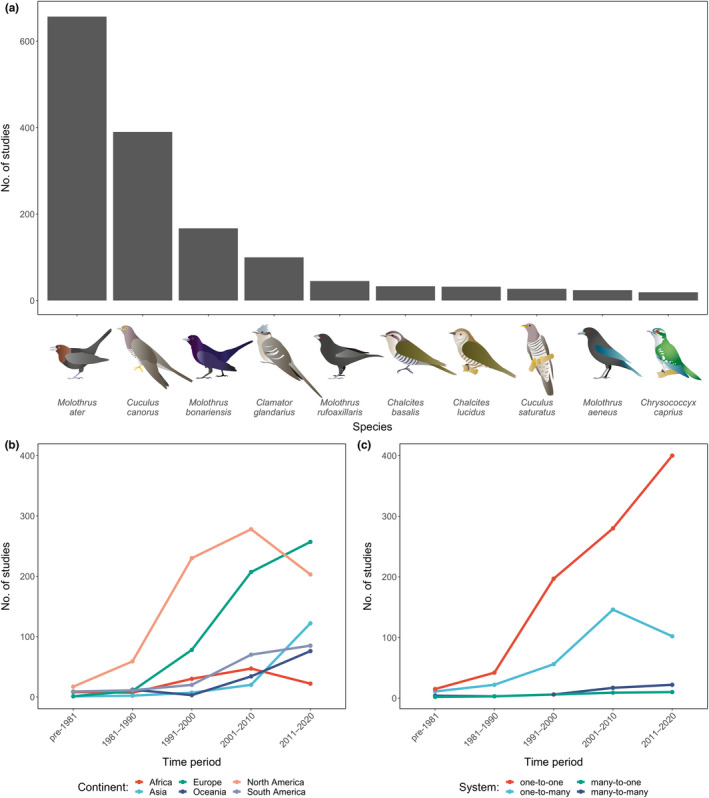
A breakdown of the number of studies: (a) investigating the 10 most frequently studied species of brood parasites; (b) by continent for each decade between 1981 and 2020, and studies pre‐1981; (c) by system type for each decade between 1981 and 2020, and studies pre‐1981. The continent of Antarctica is omitted as no brood parasites breed in the region. One‐to‐one refers to a system with one species of brood parasite and one species of host, one‐to‐many refers to a system with one species of brood parasite and multiple species of host, many‐to‐one refers to a system with multiple species of brood parasites and one species of host, and many‐to‐many refers to a system with multiple species of brood parasites and multiple species of hosts

We identified 1810 studies on avian brood parasitism published between 1934 and 2020 that met our criteria for inclusion. Approximately 75% (*n* = 1353) involved field‐based research (Table S2), with 1245 listing 644 unique field sites (Figure 2) and each study using between 1 and 17 field sites (mode = 1). Based on studies returned by *Web of Science*, we found that the number of studies increased across each consecutive decade between 1981 and 2020 (Table S3), with 70 of the world's 104 brood parasite species represented. However, the 10 most studied species all belong to either Old World cuckoos (Cuculinae) or cowbirds (*Molothrus* sp.) (Figure 3a), while the 21 species not represented in past research include 10 species of honeyguide (Indicatoridae), 10 species of Old World cuckoo and one species of cowbird (Table S1). Increases in research attention were most pronounced in Europe, with Oceania and South America receiving a steep rise in attention since 1991 and Asia since 2011 (Figure 3b). Increases were also observed in North America and Africa between 1981 and 2010; however, decreases were noted between 2011 and 2020 (Figure 3b). Correspondingly, as the geographic coverage of studies increased so did phylogenetic diversity of brood parasite study species (Figure S1). That said, most (70%, *n* = 892) field‐based research that listed field site locations were in North America and Europe (Table S4), including three studies that involved field sites in both North America and Europe. Consistent with this, over half (57%, *n* = 1025) of the studies included in our analysis focussed on the Brown‐headed Cowbird and Common Cuckoo, the two most widespread and abundant species of brood parasites in North America and Europe respectively (Figure 3a).

The majority of studies have focused on one‐to‐one systems (69%; Figure 3c; Table S5), followed by one‐to‐many systems (25%; Figure 3c; Table S5). Research investigating many‐to‐one systems, or many‐to‐many systems have received an order of magnitude less attention, accounting for 2% and 4% of studies respectively (Figure 3c; Table S5). Looking at how these patterns formed through time, one‐to‐one systems have consistently received the most research attention across the last four decades (Figure 3c). Similarly, one‐to‐many systems which received the second highest level of research attention, also have the second highest number of studies for each of the last four decades (Figure 3c). The number of studies investigating the most complex (i.e. many‐to‐many) systems increased each decade since 1981 at an approximately constant rate, such that in three of four decades these systems account for 4% of all studies, with the exception of 1991–2000 (2%). Across the last four decades, many‐to‐one systems have received little attention and have accounted for 0–4% of studies in any given decade.

### Global patterns of brood parasite–host network complexity

Of the 83 species of brood parasites with at least one documented host species, we found that they cumulatively parasitise 14% of all bird species (*n* = 1585); with 68 species of brood parasites (82%) documented as parasitising more than one species of host, and 494 species of host (31%) documented as being subject to parasitism by more than one species of brood parasite. We identified Africa as the continent with the greatest richness of brood parasites, with up to 25 species from four (out of seven) independent origins of brood parasitism (Sorenson & Payne, 2002) across three families (twice in Cuculidae [both in the sub‐family: Cuculinae], once in Indicatoridae and once in Viduidae) occurring sympatrically in sub‐Saharan Africa, especially in the vicinity of the Great Rift Valley (Figure 5a). Second to Africa, Asia is home to hotspots of brood parasite species richness where up to 16 species of Old World cuckoos, from two independent origins of brood parasitism, occur in sympatry through the Himalayan Range and especially the Eastern Himalayas, as well as the Malay Peninsula and Greater Sundas. Note that the honeyguides in this region were not included as they have no known hosts. In Oceania, brood parasite species richness is greatest in northern Australia and East of the Great Dividing Range, where up to 10 species of Old World cuckoos from a single lineage occur sympatrically. Brood parasite species diversity is low throughout Europe with only three species from two lineages (both Old World cuckoos) present: the Common, Oriental (*Cuculus saturatus*) and Great Spotted Cuckoos (*Clamator glandarius*). Similarly, much of North America has low brood parasite species diversity where the Brown‐headed Cowbird is the only species present through much of the Nearctic. Across the whole of the Americas, the Central Andes and the upper Paraná River basin have the greatest diversity of brood parasites with up to six species from three brood parasitic lineages (Anatidae, Cuculidae [sub‐family: Neomorphinae] and Icteridae) found sympatrically.

Similar to brood parasite species richness, host species richness in Africa is greatest in the Great Rift Valley, especially the southern part of the Gregory Rift and the Albertine Rift, where up to 188 host species (44 families) occur in sympatry (Figure 5b). A comparable pattern is seen in Asia where host species richness is greatest in the Eastern Himalayas, where up to 184 host species (48 families) occur in sympatry. For much of the rest of the world, however, we found that global patterns of host species richness appear not to strongly co‐vary with patterns of brood parasite species richness with relatively low numbers of host species residing in the Malay Peninsula and Greater Sundas (up to 78 host species across 34 families occurring in sympatry) despite being a hotspot for brood parasite species diversity with up to 12 species occurring in sympatry. While northern Australia has relatively few host species, South‐East Australia and the region East of the Great Dividing Range has high host diversity with up to 126 species (31 families) occurring in sympatry. Regions with relatively low brood parasite species diversity such as the Americas have relatively high host diversity with up to 114 (33 families) and 141 (30 families) species occurring in sympatry in parts of North and South America respectively. In Europe, the greatest host diversity is found in the northern Mediterranean where up to 89 host species (30 families) occur in sympatry.

**FIGURE 4:**
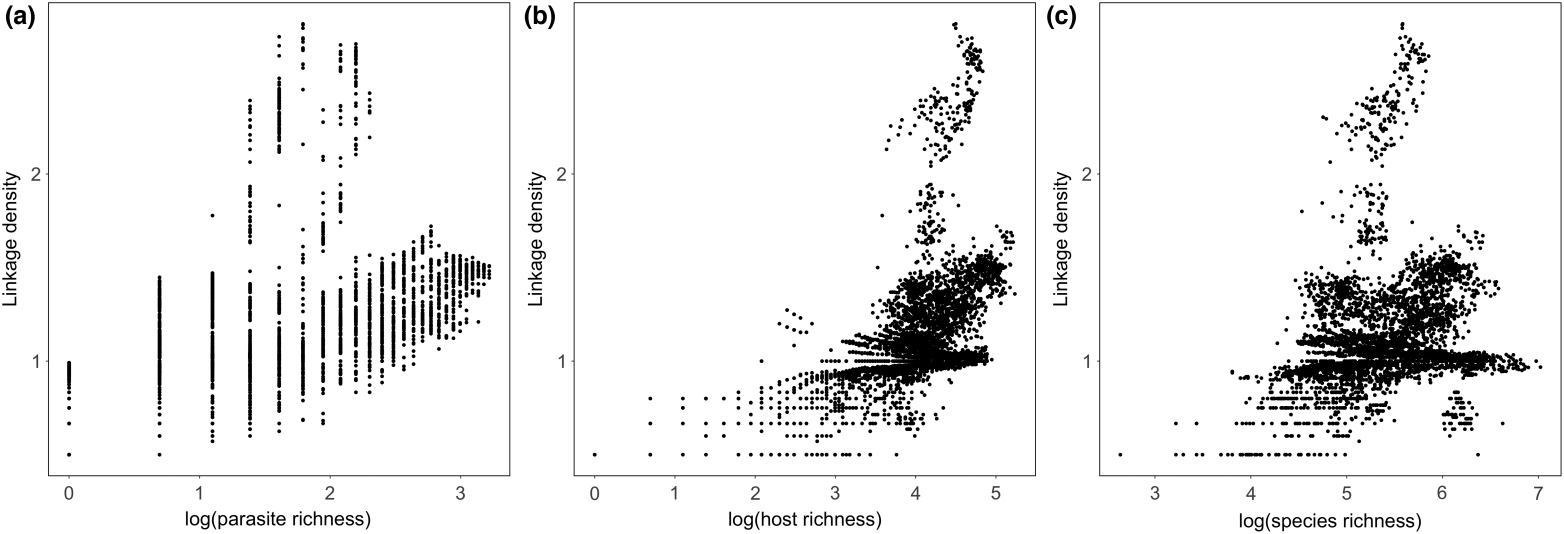
Plots describing the relationship between system complexity measured as the linkage density of potential brood parasite–host systems for each respective land hexagon, and three metrics of species richness: (a) number of species of brood parasites; (b) number of species of hosts; (c) number of bird species. Each dot represents one land hexagon

**FIGURE 5:**
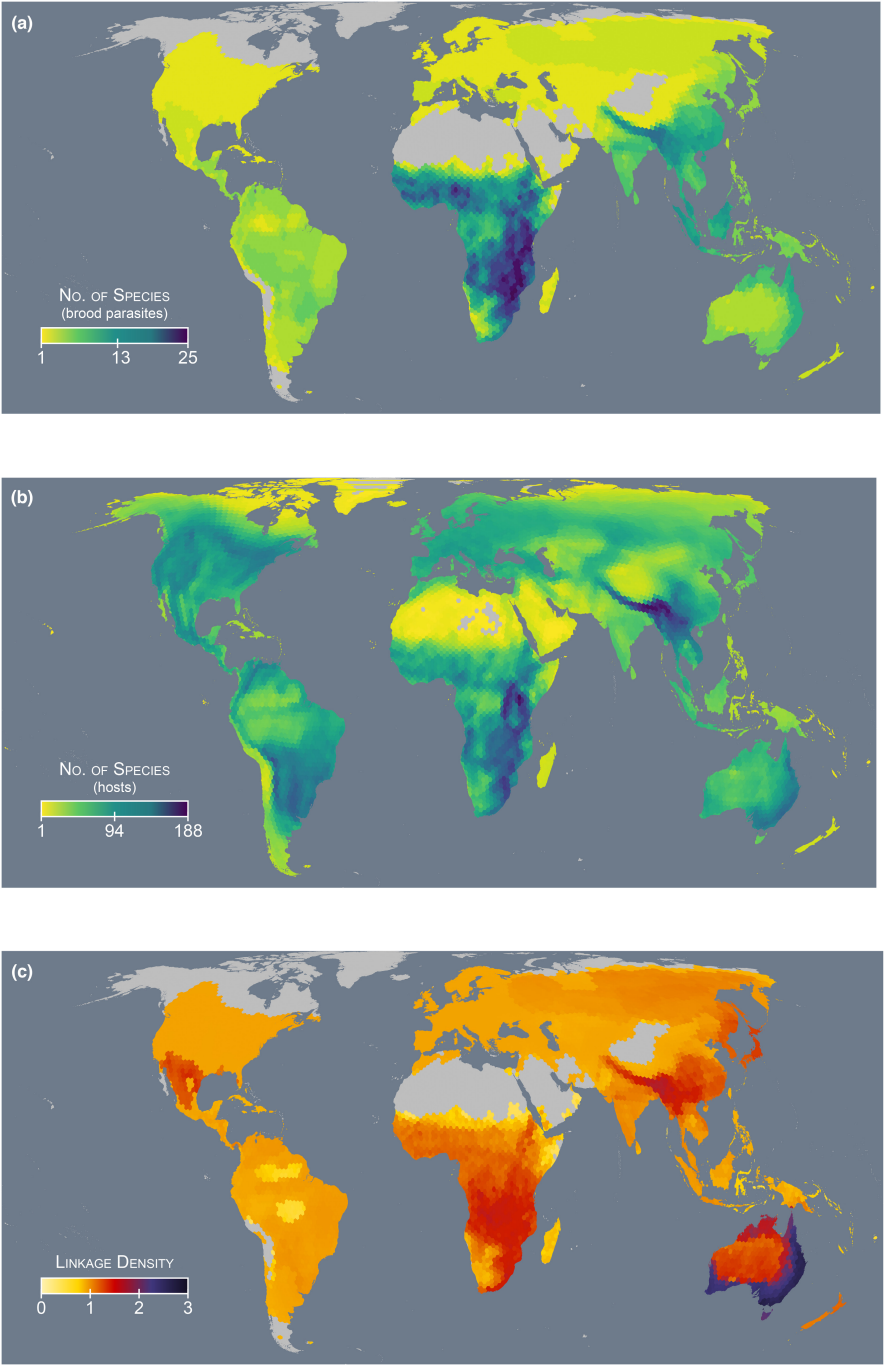
Heatmaps describing (a) global patterns of brood parasite species diversity; (b) host species diversity; and (c) brood parasite–host system complexity. System complexity is measured as the linkage density of potential brood parasite–host systems for each respective land hexagon. Grey land areas represent regions where either no species of brood parasites (a and c) or hosts (b) occur

Global patterns of linkage density (Figure 5c), our measure of network complexity, is significantly and positively associated with brood parasite species richness (OLS: *R* ^2^ = 0.24, *F*[1, 5570] = 1717, *p* < 0.001; SAR: Nagelkerke's *R* ^2^ = 0.95, LR = 1781, df = 1, *p* < 0.001; Figures 4a and 5a), host species richness (OLS: *R* ^2^ = 0.19, *F*[1, 5570] = 1291, *p* < 0.001; SAR: Nagelkerke's *R* ^2^ = 0.95, LR = 1479, df = 1, *p* < 0.001; Figures 4b and 5b) and overall bird species richness (OLS: *R* ^2^ = 0.05, *F*[1, 5570] = 300, *p* < 0.001; SAR: Nagelkerke's *R* ^2^ = 0.94, LR = 1165, df = 1, *p* < 0.001; Figure 4c). Results for OLS and SAR models are qualitatively similar in terms of significance and slope estimate. For the SAR models, the autocorrelation term explained most of the variation in the residuals, creating very high *R* ^2^ values that are not informative; while for the OLS models, the *R* ^2^ values are moderate for brood parasite and host species richness and low for overall species richness, thus indicating that network complexity is not a simple function of the distribution of avian species including brood parasites and hosts. We found that linkage density ranged from 0.5 (i.e. one brood parasite and one host with one interaction) up to 2.8 (i.e. six brood parasites and 80 hosts with 241 interactions) and that South‐East Australia and the region East of the Great Dividing Range, is the global hotspot of brood parasite–host network complexity. By contrast, the regions with the greatest diversity of brood parasites and hosts in sub‐Saharan Africa and the Himalayas are home to networks with maximum values of 1.6 and 1.7 respectively. While Europe and the Americas have moderate host richness (Figure 5b), low brood parasite species diversity means that network linkage densities are inherently low across much of these regions (maximum of 1.1 in Europe, 1.5 in North America and 1.1 in South America; Figure 5c) with little geographic variation, thus mirroring patterns seen in brood parasite species richness (Figure 5a).

## DISCUSSION

Most of the world's brood parasites (83% of those with at least one known host species) have been recorded to parasitise at least two species of host and 31% of recorded hosts have been parasitised by at least two species of brood parasite, with the most complex network comprising 241 interactions. This contrasts with the majority of past research (69%) that has focussed on understanding the ecology and evolution of pairwise interactions. Furthermore, most studies have focused on North American and European study systems (70%; Figures 2, 3; Table S4) and, along with South America (another 10%), these regions are notable in that they are home to relatively simple brood parasite–host interaction networks compared to their analogues in Africa, Asia and Oceania (Figure 5c). Taken together, these data indicate that brood parasite–host interactions are more complex than they have generally been portrayed in the literature, and that the geographic biases of past research efforts may, at least partially, explain the disconnect between research efforts and a proportionate examination of these systems' complexity. Given that these more complex scenarios are prevalent globally (Figure 5c), and that other comparable fields of research have yielded major new insights through the analysis of study systems in a multispecies context (indirect effects in mutualistic networks, Guimarães et al., 2017; rapid molecular evolution in bacterium parasitised by viral parasites, Betts et al., 2018; coevolution shaping morphology in plant–pollinator mutualisms Lomáscolo et al., 2019), we suggest that the examination of more complex brood parasite–host relationships may offer new opportunities to explore how multispecies interactions affect eco‐evolutionary processes and shape biodiversity.

### Patterns of past research

With the majority of brood parasitism publications resulting from work conducted in North America and Europe (Figures 2, 3; Table S4), this geographic bias is similar to that seen in the wider ecology literature (Amano et al., 2021; Martin et al., 2012; Nuñez et al., 2019, 2021). Furthermore, we found that once a field site was established, it often became the subject of further brood parasitism studies (Figure 2). This said, over the last three decades, an increasing proportion of brood parasitism research has been conducted in Asia, Oceania and South America (Figure 3b) which corresponds with the phylogenetic diversity of studied species also increasing during this period (Figure S1). Another factor that may influence research emphasis is the potential impact that a brood parasite has on the populations and demography of its hosts and, in turn, conservation policies and management actions. This factor is almost exclusive to the Brown‐headed Cowbird, which has been the subject of a plethora of studies focussing on the conservation implications of parasitism especially on currently or formerly endangered species (Cooper et al., 2019; Hauser et al., 2019). Brood parasitism has also been identified as a threat to at least one host of the Common Cuckoo (Zhang et al., 2020) and several hosts of the Shiny Cowbird (*Molothrus bonariensis*) (e.g. Atencio et al., 2020).

The examination of relatively simple (i.e. one‐to‐one) networks has accounted for the majority of brood parasitism research, including in areas where more complex systems are the norm (e.g. Africa, Asia and Oceania) despite increasing research efforts in these regions (Figure 3c; Table S5). We suggest two primary reasons that may help to explain this pattern. First, this emphasis was likely shaped by early work such as Rothstein's (1990) seminal review, which proposed brood parasitism as a model system for studying coevolution. This review noted that most hosts are parasitised by a single species of brood parasite, which may have contributed to a tradition of studying one‐to‐one exchanges (e.g. Duffy et al., 2021). While it is true that the majority (69%) of documented hosts are reportedly parasitised by a single species of brood parasite, an even greater majority (83%) of brood parasite species parasitise more than one species of host. This pattern of parasites impacting more than one host species is to be expected given that brood parasites require hosts to raise their offspring and hosts evolve adaptations to deter or evade parasitism that can make them no longer suitable as a host (Langmore et al., 2005). Thus, while coevolutionary processes might exclusively shape reciprocal phenotype evolution in some areas, we should equally expect that adaptations in other areas could be influenced by interactions with sympatric brood parasites and/or hosts. For example, in a many‐to‐many system with brood parasites that have highly mimetic eggs, host species partition egg phenotypic space more distinctly than related non‐parasitised species, in order to evade parasitism by multiple brood parasite egg phenotypes (Caves et al., 2017). Another reason that may help explain this pattern is the general difficulty of comprehensively monitoring the nests of bird populations in the field, hence studying one‐to‐one systems where nests of only one species of host need to be monitored has likely shaped their prevalence in the literature. While difficult to quantify, this is particularly evident for studies involving the detailed examination of the same host populations over multiple years, such as the Common Reed‐warblers (*Acrocephalus scirpaceus*) at Wicken Fen in the United Kingdom (Davies, 2000; Stoddard & Kilner, 2013) or the Great Reed‐warblers (*A. arundinaceus*) near Apaj village in Hungary (Geltsch et al., 2017; Hauber et al., 2021).

### Global patterns of brood parasite–host network complexity

Despite having fewer brood parasite species than sub‐Saharan Africa and the Himalayas, we found Australia to have the highest network complexity in the world (Figure 5c). A possible explanation for more complex networks being found in Australia, is that Australia's 10 brood parasites are all Old World cuckoos from a single lineage so share a broadly similar physiology and behavioural repertoire which may have led to a common compatibility with host species that share particular characteristics (Brooker & Brooker, 1989). By contrast, sub‐Saharan Africa, is home to 39 species of brood parasites from three families: Indicatoridae (seven species, excluding eight others with no known hosts), Viduidae (viduid finches, 20 species), and Cuculidae (from two lineages within Cuculinae, 12 species, excluding one with no known hosts) (Table S1). While cuckoos parasitise a range of open and dome nesting passerine species, honeyguides favour cavity nesters that are not parasitised by either cuckoos or viduid finches. With the exception of the Cuckoo‐finch (*Anomalospiza imberbis*), viduid finches specialise on estrildid finches (Estrildidae), which are not parasitised by cuckoos or honeyguides. This is possibly related to the granivorous diet of nestling estrildid finches (Jamie et al., 2021) compared to the typically insectivorous diet of cuckoos and honeyguides (Morelli et al., 2020; Short & Horne, 1985). Furthermore, there is little overlap in host species among *Vidua* species as speciation among members of this genus has been facilitated by host specialisation (Jamie et al., 2021; Sorenson et al., 2003). With four lineages of brood parasites across three avian families, and their vastly different biologies, the result is that Africa is home to more fragmented networks with on average lower values of network linkage density than Australia. However, phylogenetic diversity across brood parasites does not explain the differences in system complexity between Australia and the Himalayas, where brood parasites included to calculate linkage density all belong to two lineages within Cuculinae. Note that the one species of honeyguide that occurs in the Himalayas was excluded as it has no known hosts.

With brood parasite–host network complexity strongly and positively correlated with brood parasite species richness, host species richness and overall bird species richness, the regions that host the greatest diversity of brood parasites and hosts and the most complex networks, are principally found at lower latitudes in the tropics and sub‐tropics. The remarkable exception to this pattern is in the Neotropics; despite being the region with the greatest avian diversity on Earth (Pillay et al., 2021), it is home to relatively few brood parasite and host species (Figure 5). This is likely associated with the species‐poor parasitic lineages found in the region, as well as the lack of cooperative breeding species which are commonly hosts of brood parasites and whose presence is positively correlated with that of brood parasites (Feeney et al., 2013). If brood parasites selectively target cooperatively breeding species, this could lead to more complex networks as brood parasites compete for access to nests of the most suitable hosts. This may provide an explanation for why we find more complex brood parasite–host networks in Australia (Figure 5c) where there is a greater richness of cooperatively breeding species, compared to the Himalayas which hosts a lower diversity of cooperatively breeding species.

It is also noteworthy that the hotspots of global network complexity tend to coincide with tropical and sub‐tropical montane regions which are associated with high species richness and endemism (Myers et al., 2000; Rahbek et al., 2019). A wide range of interacting factors contribute to the diversity of these regions including the topographical complexity of tropical mountain ranges which typically harbour a wide spectrum of temperature–precipitation climate space within a relatively small area (Rahbek et al., 2007, 2019), and ecological limits through niche packing and higher resource diversity contribute to high biodiversity, especially at mid‐elevations (Beck et al., 2017; Schumm et al., 2020). With respect to brood parasitism, one may expect that high host species diversity in montane regions and niche packing due to abiotic (e.g. temperature) and biotic (e.g. diet) factors could have led to the evolution of a diversity of brood parasites that have little niche overlap including in host preferences, however, we find that the opposite appears to hold with highly connected networks found in these regions (Figure 5c). A possible explanation is that temperature and habitat are more important determinants of species' distributions than competition (Elsen et al., 2017). This could mean that temperature and habitat availability are stronger drivers of niche packing in brood parasites than host choice; so, given a narrow elevational window of suitable climatic conditions, brood parasites may benefit from targeting a variety of host species.

With aspects of the natural history of Australia's birds being better known than sub‐Saharan Africa and eastern Asia (Xiao et al., 2017), which may be related to geographical sampling biases (Hughes et al., 2021), it is also important to consider whether gaps in our knowledge of African and Asian brood parasites may have contributed to our findings. This is exemplified by there being no known hosts for 15 species of African and Asian brood parasites (Table S1). With more thorough knowledge of the hosts of African and Asian brood parasites, it is possible that brood parasite–host networks across these regions are more complex and interconnected than they appear. To account for the effect of knowledge biases, a correction factor could be applied. We suggest that because museum egg collections may provide the most accurate reflection of our knowledge of which species are hosts to a brood parasite, the examination of biases in collections may be a potential route to compute such a correction factor.

Finally, despite being the areas with the most studies, North America and Europe are among the regions with the lowest brood parasite–host system complexity. This is principally because no more than three species of brood parasites occur sympatrically in each of these regions, and despite the two most ubiquitous species in each respective continent having hundreds of documented hosts (Lowther, 2019d, b), there is generally little overlap in host species between different species of sympatric brood parasites. However, where two species (Brown‐headed and Bronzed Cowbirds *Molothrus aeneus*) of closely related host‐generalists occur in sympatry in northern Mexico and the southern USA, through the Sierra Madre Occidental and Sierra Madre Oriental (Figure 5a), we find the greatest levels of system complexity in the Americas (Figure 5c). Interestingly, the Common Cuckoo, and Brown‐headed and Shiny Cowbirds are the three species of brood parasites with the most documented host species (Figure 3a). While geography (Hughes et al., 2021; Nuñez et al., 2019, 2021) may have favoured the discovery of hosts for these three species, it is also possible that each of these host‐generalists have benefitted from a lack of competition for host species due to the dearth of brood parasite species diversity in Europe and the Americas. Importantly, that these three brood parasites comprise several subspecies (Handbook of the Birds of the World and BirdLife International, 2019), and the Common Cuckoo also exhibits numerous host‐specific races (or *gentes*)—distinct evolutionary lineages that specialise on specific host species, or groups of host species (Fossøy et al., 2016; Gibbs et al., 2000)—has not been considered in our analysis, and their consideration may result in different patterns of network complexity than that which we have presented. To explore this further, future research could investigate geographic variation in host‐use for each of these, and other, polytypic brood parasite lineages. Likewise, recorded hosts are not used equally by brood parasites, and there are several species that are only rarely recorded as hosts. In some cases, these species are thought to be unsuitable as hosts due to greatly differing ecologies and life histories than those required by brood parasites and may have been parasitised accidentally (Rutila et al., 2002), while others frequently reject brood parasite eggs or chicks which may have led to them being rarely targeted as a host (Moksnes & Røskaft, 1992). Due to knowledge biases towards certain brood parasite species and particular regions (Figures 2, 3), we were not able to consider these nuances in our analysis. Nonetheless, we emphasise their potential importance in future work to gain a more complete understanding of network complexity that exists between brood parasite species, subspecies and *gentes*, and their hosts; as well as the role that the frequency of host‐use plays in shaping the evolutionary trajectories of relationships with brood parasites.

### Multispecies interactions

The archetypal image of a brood parasite is of one locked in a high‐stakes arms race with its host, where directional, stabilising and diversifying selection produces increasingly refined coevolved adaptations and counter‐adaptations across all stages of the host's nesting cycle (Table 1; Davies, 2011). While this may be true of some species, this unidimensional portrayal is not true of most. We found that 83% of the world's brood parasites have at least two host species, with 11 (IQR = 24) being the median number of host species for brood parasites and some species having as many as 274 hosts as in the case of the Shiny Cowbird. Correspondingly, 31% of host species are known to be parasitised by at least two and up to eight species of brood parasites in the case of Silvereye (*Zosterops lateralis*). Thus, while the use of a pairwise framework has led to numerous major breakthroughs in our knowledge of brood parasitism and coevolution, there is a notable lack of examination of these globally common multispecies scenarios.

**TABLE 1:** Types of selection responsible for driving evolution in brood parasites and hosts in response to different brood parasite–host interaction network scenarios

Network type	Brood parasite		Host	
	Pre‐parasitism	Post‐parasitism	Pre‐parasitism	Post‐parasitism
One‐to‐one	Coevolution between one brood parasite and one host. This widely investigated and discussed field includes classic examples of directional selection, such as increasing levels of pattern complexity of brood parasite and host egg phenotypes; stabilising selection, such as for visual and acoustic predatory‐hawk mimicry by brood parasites to evade detection by hosts; and diversifying selection, such as the evolution of eggshell colour polymorphisms among brood parasites and hosts			
One‐to‐many	Stabilising selection, such as for cryptic behaviours in brood parasites to avoid detection by multiple host species simultaneously and enable egg‐laying without detection or injury.		Directional selection, such as for hosts eavesdropping on acoustic signals by other host species which could lead to commensal or mutualistic relationships between host species	
Many‐to‐one	Competition for access to, and occupation of, host nests by multiple brood parasite species leading to directional selection, such as for earlier hatching to monopolise provisioning.		Stabilising selection towards phenotypes that defend against all brood parasite species such as hosts mobbing adult brood parasites and an optimal egg phenotype in hosts to enable discrimination of eggs from multiple brood parasite species	
Many‐to‐many	Competition for access to multiple host species, leading to directional selection and high rates of phenotypic evolution in brood parasites	Stabilising selection for egg and nestling phenotypes in brood parasites that enables exploitation of multiple host species	Stabilising selection for pre‐parasitism defences could lead to convergent evolution across multiple host species for an optimal defence strategy	Diversifying selection by multiple host species for phenotypic traits to reduce mimicry across multiple parasite species

*Note*: Four types of network are considered: One‐to‐one (i.e. one brood parasite and one host), one‐to‐many, many‐to‐one and many‐to‐many. Along with three types of selection: Directional, stabilising and diversifying. Adaptations that occur prior to the parasite laying its egg in the host nest can be influenced by interactions with secondary host or brood parasite species (Feeney, 2017), but this is less often the case in interactions in the egg, nestling and fledgling stages of the nesting cycle. As such, we suspect that evolutionary processes operate in a broadly similar manner following parasitism, which may differ to those prior to parasitism occurring.

Examining the ecology and evolution of brood parasites and hosts through a multispecies, rather than as strictly pairwise, framework may have implications for our predictions about how selection might operate across different stages of the nesting cycle. For example, directional selection has been documented in one‐to‐one systems, such as the Common Cuckoo, which has evolved egg phenotypes to mimic the eggs of their Great Reed‐warbler hosts so that the majority of their eggs are not recognised and ejected from the nest by the warbler foster parents. In response, the warbler has evolved increasingly complex egg patterns as the two species escalate the evolutionary arms‐race (Geltsch et al., 2017); however, some cases of directional selection would be missed without considering interactions beyond the pairwise framework (Table 1). For instance, in the Red‐winged Blackbird (*Agelaius phoeniceus*), a host of the Brown‐headed Cowbird, there is evidence of directional selection for them to eavesdrop on the ‘*seet’* vocalisation by Yellow Warblers (*Setophaga petechia*), which signals the presence of a brood parasite (Lawson et al., 2020). This adaptation represents an example of the emergence of a commensal relationship between host species, as Red‐winged Blackbirds utilise the Yellow Warbler referential alarm call to elevate their frontline defences against a danger to their nest. In turn, the Yellow Warbler experiences reduced rates of parasitism by Brown‐headed Cowbirds when nesting near Red‐winged Blackbirds, as they benefit from Red‐winged Blackbirds aggressively mobbing Brown‐headed Cowbirds (Clark & Robertson, 1979), which indicates the possible emergence of a mutualistic relationship. In a scenario where multiple species of brood parasites are competing for access to the same species of host, we may also expect to see Red Queen dynamics operate across the brood parasite community, such that multiple species may evolve adaptations and counter‐adaptations to outcompete each other, such as having a shorter incubation period to hatch first and monopolise foster parental provisioning (Flower et al., 2015).

Where one species of brood parasite parasitises multiple host species we see stabilising selection for adaptations that collectively impact the host community, such as secretive behaviours to avoid detection by multiple host species (Thorogood & Davies, 2016; York & Davies, 2017). In response, there is evidence of selection for acoustically similar context‐specific alarm calls which indicate the presence of a brood parasite to conspecifics and possibly heterospecifics (Feeney, 2017; Wheatcroft & Price, 2015). Examining the extent to which such a vocalisation has been selected for across the host community presents an enticing opportunity to identify an example of convergence on a defensive phenotype across multiple host species. Such a behaviour would also represent an example of stabilising selection and the evolution of mutualistic interactions between host species which would, in turn, reinforce (stabilising) selection for this behavioural phenotype.

Diversifying selection sometimes in combination with negative‐frequency dependent selection is also apparent in one‐to‐one systems. For instance, the Tawny‐flanked Prinia (*Prinia subflava*) has been shown to increase egg colour diversity over time (Spottiswoode & Stevens, 2012) which increases the ability of Tawny‐flanked Prinias to reject eggs of the brood parasitic Cuckoo‐finch, as the colour of the brood parasite's eggs is less likely to match that of the host allowing for easier discrimination (Stevens et al., 2013). In response, the Cuckoo‐finch has increased its egg colour diversity over the same period (Spottiswoode & Stevens, 2012). Caves et al. (2017) extended on these studies, and showed that sympatric cisticolid (Cisticolidae) hosts of the brood parasitic Cuckoo‐finch, which includes the Tawny‐flanked Prinia, exhibit greater partitioning in colour space compared to non‐hosts. By considering multiple host species of the Cuckoo‐finch, it has been shown that diversifying selection by brood parasites can shape the evolution of egg phenotypes across entire host communities. With interspecific competition among brood parasites for access to hosts, this may have also led to high rates of evolution in plumage and egg size of cuckoos (Medina & Langmore, 2015). The examination of how egg phenotypes of both brood parasites and hosts evolve under circumstances where multiple brood parasites compete for access to nests of multiple host species remains unstudied, but represents an exciting prospect for future work.

Interactions between sympatric brood parasites and hosts can be complex, however, they only represent a subset of the ecological interactions that these organisms experience (Pollock et al., 2021). As such, insights may be gained from research on other systems in which multiple sources of selection shape the ecology and evolution of the involved species. For example, Ramos and Schiestl (2019) found that the evolution of traits in plants was driven by mutualistic interactions with bee pollinators and antagonistic interactions with herbivores. Specifically, plants under selection by bee pollinators evolved greater floral attractiveness, which was not the case for plants also exposed to herbivory as they instead evolved higher degrees of self‐compatibility. Within the brood parasitism literature, there has been some interest in the influence that additional interactions have on brood parasite–host relationships. For instance, interactions with predators can drastically influence how hosts defend themselves against brood parasitism (Krüger, 2011) and determine whether hosting a brood parasite imposes a cost, or offers a benefit to the host's fitness (Canestrari et al., 2014).

## CONCLUDING REMARKS

The interactions between brood parasites and hosts are powerful and enduring models for exploring how eco‐evolutionary processes shape the evolution of phenotypes within, and patterns across species (Feeney et al., 2014; Moksnes et al., 2013; Stoddard & Hauber, 2017). Our work here shows that most of the world's brood parasites interact with multiple species of hosts and global patterns of network complexity are highly heterogeneous. Thus, in addition to being used to study interactions between pairs of species in the manner that has overwhelmingly characterised past research efforts, these systems also provide potentially fruitful, yet largely unexplored, opportunities to study the ecology and evolution of multispecies interactions in a manner that has become commonplace in other similar fields of research (Afkhami et al., 2014; Burmølle et al., 2014). While complex networks are found globally, we suggest that the hotspots of network complexity we have identified in Australia, the Himalayas, sub‐Saharan Africa and northern Mexico and the southern United States present the best opportunities for the study of multispecies interactions. Furthermore, while we have focused on the potential opportunities offered by studying multispecies interactions between brood parasites and hosts, these interactions represent only a subset of the interactions that can influence the evolutionary outcomes of these exchanges (Canestrari et al., 2014; Krüger, 2007). Ultimately, we suggest that the incorporation of additional layers of complexity into the study of brood parasitism will open a new set of frontiers for our understanding of the ecological and evolutionary processes that have shaped brood parasitism and the world's biodiversity.

## AUTHORSHIP

J.A.K., M.S., A.M. and W.E.F. conceived and designed the study. J.A.K. collated data on documented brood parasite–host relationships. J.A.K., N.M.R., M.E.H. and W.E.F. contributed to data extraction following the literature search. J.A.K, M.S. and A.M. conducted data analysis. J.A.K and W.E.F. wrote the manuscript with input from all authors.

### OPEN RESEARCH BADGES

This article has earned Open Data and Open Materials badges. Data and materials are available at [https://datadryad.org/stash/share/W3B56MXJtyG90YbZQofScIAvqshl3px6soU2nM_d0r4].

### PEER REVIEW

The peer review history for this article is available at https://publons.com/publon/10.1111/ele.14062.

## Supporting information


Table S1
Table S2
Table S3
Table S4
Table S5
Figure S1

## Data Availability

All data used for this paper are archived in the Dryad data repository (https://doi.org/10.5061/dryad.7wm37pvv4).
