# Supplementary material for: The overlooked complexity of avian brood parasite–host relationships
Source: Ecol Lett. 2022 Jun 28;25(8):1889–904. doi: 10.1111/ele.14062 (PMC9543277; doi:10.1111/ele.14062)
Supplement: Supplementary file 1 — Table S1 Table S2 Table S3 Table S4 Table S5 Figure S1 [file ELE-25-1889-s001.docx]

**SUPPLEMENTARY MATERIALS**

**Table S1.** Brood parasite species with no documented hosts so were excluded from further analyses. Names based on Handbook of the Birds of the World and BirdLife International (2019) taxonomy.

| **Vernacular name** | **Scientific name** |
| --- | --- |
| Dwarf Koel | *Microdynamis parva* |
| Long-billed Cuckoo | *Chalcites megarhynchus* |
| Rufous-throated Bronze-cuckoo | *Chalcites ruficollis* |
| White-eared Bronze-cuckoo | *Chalcites meyerii* |
| White-crowned Cuckoo | *Caliechthrus leucolophus* |
| Olive Long-tailed Cuckoo | *Cercococcyx olivinus* |
| Philippine Drongo-cuckoo | *Surniculus velutinus* |
| Moluccan Drongo-cuckoo | *Surniculus musschenbroeki* |
| Philippine Hawk-cuckoo | *Hierococcyx pectoralis* |
| Sulawesi Cuckoo | *Cuculus crassirostris* |
| Cassin’s Honeybird | *Prodotiscus insignis* |
| Zenker’s Honeyguide | *Melignomon zenkeri* |
| Yellow-footed Honeyguide | *Melignomon* *eisentrauti* |
| Dwarf Honeyguide | *Indicator pumilio* |
| Willcock’s Honeyguide | *Indicator willcocksi* |
| Least Honeyguide | *Indicator exilis* |
| Spotted Honeyguide | *Indicator maculatus* |
| Yellow-rumped Honeyguide | *Indicator xanthonotus* |
| Malay Honeyguide | *Indicator archipelagicus* |
| Lyre-tailed Honeyguide | *Melichneutes robustus* |
| Bronze-brown Cowbird | *Molothrus armenti* |

**Table S2.** The number of studies assigned to each of the six categories of research recognised by this study.

| **Study category** | **Number of studies** |
| --- | --- |
| Field-based | 1,353 |
| Review and/or meta-analysis | 278 |
| Theoretical and/or computational | 76 |
| Laboratory-based | 52 |
| Museum and/or collection-based | 35 |
| Molecular and/or genomic | 16 |

**Table S3.** Breakdown of the number of studies investigating obligate avian brood parasitism by decade based on a literature search covering 1912–2020.

| **Time period** | **Number of studies** |
| --- | --- |
| pre-1981 | 32 |
| 1981–1990 | 70 |
| 1991–2000 | 259 |
| 2001–2010 | 452 |
| 2011–2020 | 534 |

**Table S4.** Breakdown of the number of studies investigating obligate avian brood parasitism by continent based on a literature search covering 1912–2020. Antarctica is omitted as no brood parasites breed on the continent.

| **Region** | **Number of studies** |
| --- | --- |
| North America | 787 |
| Europe | 553 |
| South America | 195 |
| Asia | 152 |
| Oceania | 125 |
| Africa | 114 |

**Table S5.** Breakdown of the number of studies investigating obligate avian brood parasitism by system type based on a literature search covering 1912–2020. One-to-one refers to a system with one species of brood parasite and one species of host, one-to-many refers to a system with one species of brood parasite and multiple species of host, many-to-one refers to a system with multiple species of brood parasites and one species of host, and many-to-many refers to a system with multiple species of brood parasites and multiple species of hosts.

| **System type** | **Number of studies** |
| --- | --- |
| One-to-one | 934 |
| One-to-many | 337 |
| Many-to-one | 24 |
| Many-to-many | 52 |


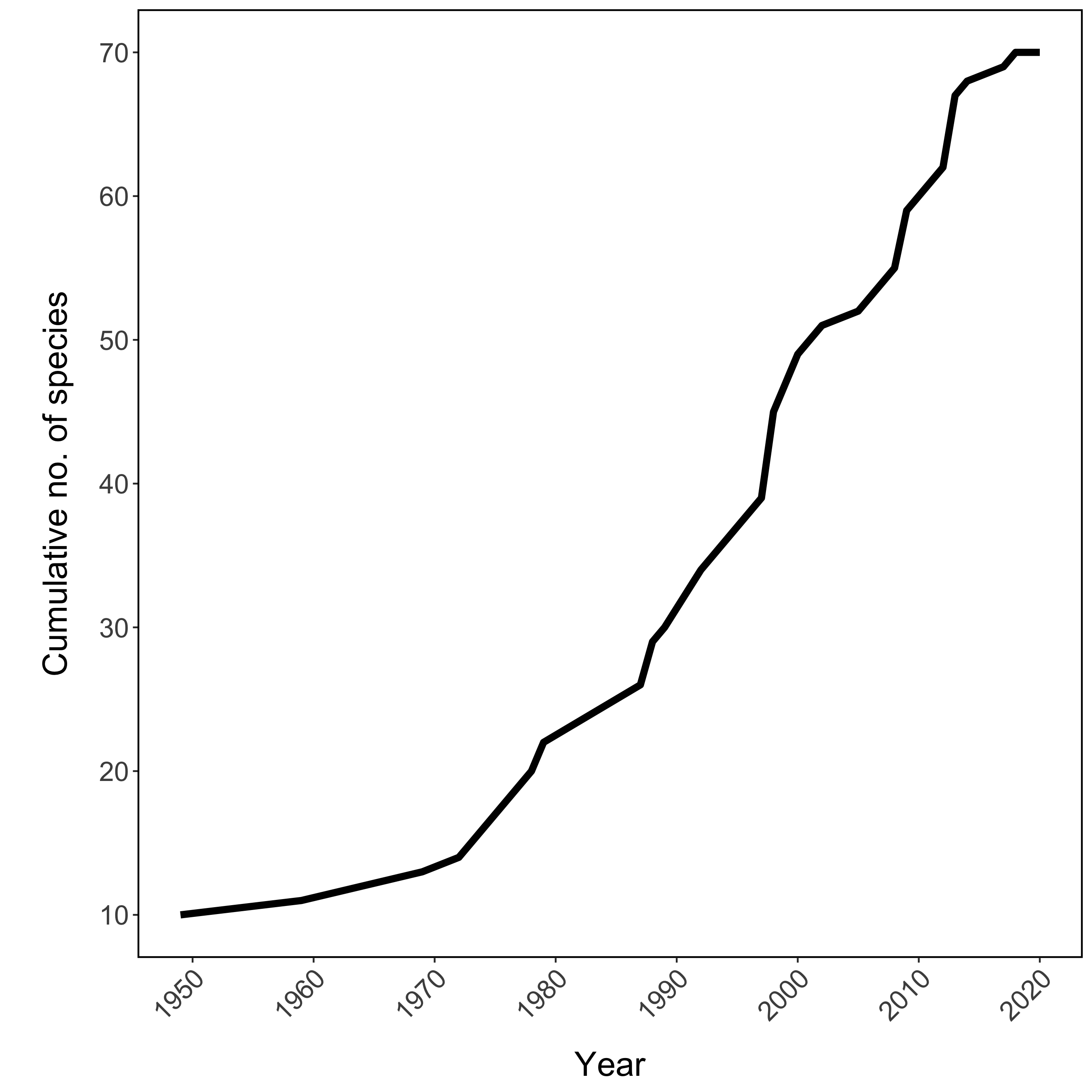


**Figure S1.** The cumulative number of brood parasite species that have been study species, between 1948 and 2020; based on the year the publications when each species first appeared as a focal study species.
